# Prevalence of stillbirth at the Buea Regional Hospital, Fako Division south-west region, Cameroon

**DOI:** 10.11604/pamj.2019.33.315.17979

**Published:** 2019-08-21

**Authors:** Nkengafac Boris Anu, Claude Ngwayu Nkfusai, Marvelle Nanyongo Mbua Evelle, Liza Enanga Efande, Fala Bede, Joyce Shirinde, Samuel Nambile Cumber

**Affiliations:** 1 Department of Nursing, Faculty of Health Sciences, University of Buea, Buea, Cameroon; 2 Department of Microbiology and Parasitology, Faculty of Science, University of Buea, Buea, Cameroon; 3 Cameroon Baptist Convention Health Services, Yaounde, Cameroon; 4 School of Health Systems and Public Health, Faculty of Health Sciences, University of Pretoria Private Bag X323, Gezina, 0001, Pretoria, South Africa; 5 Faculty of Health Sciences, University of the Free State, Bloemfontein, South Africa; 6 Section for Epidemiology and Social Medicine, Department of Public Health, Institute of Medicine (EPSO), The Sahlgrenska Academy at University of Gothenburg, Gothenburg, Sweden

**Keywords:** Stillbirths, prevalence, gender, abortion, deliveries, antenatal clinics, meme division, south west region, Cameroon

## Abstract

**Introduction:**

The study investigated the prevalence of stillbirth at the Buea regional hospital, by taking cases of pregnant women who attended antenatal clinic(s) and those who did not attend but had their deliveries at the Buea regional hospital. The study specifically estimated the prevalence of stillbirths; identified possible risk factors associated with stillbirths, and determined whether the number of antenatal clinic visits is related to the occurrence of stillbirths-because during antenatal clinic visits, pregnant women are educated on risk factors of stillbirths such as: preterm deliveries; sex of the stillbirth; history of stillbirth; history of abortion(s); what age group of mothers are more likely to have a stillbirth.

**Methods:**

The study was a hospital based retrospective study at the maternity in which there were 3577 deliveries registered at the Buea regional hospital dated May 1^st^, 2014 to April 30^th^, 2017. With the aid of a checklist data was collected, analysed and presented with the use of tables, pie-charts and bar charts.

**Results:**

The prevalence of stillbirths was 26‰; possible risk factors associated with stillbirths included: preterm deliveries; women aged 20-29 years; history of abortion(s); a history of stillbirth; sex of stillbirths were more of females than males; and insufficient antenatal clinic attendance (≤1 antenatal clinic attendance) had more stillbirths.

**Conclusion:**

The study established that stillbirths can occur in any woman of child-bearing age. possible risk factors associated with stillbirths included: preterm deliveries; women aged 20-29 years; history of abortion(s); a history of stillbirth; gender of stillbirths were more of females than males; and insufficient antenatal clinic attendance (≤1 antenatal clinic attendance) had more stillbirths.

## Introduction

Stillbirth is defined as “a baby born with no signs of life at or after 28 weeks of gestation” [1]. It is also defined as the delivery of a dead foetus whose birth weight is more than 500g [2]. One hundred years ago, stillbirth rates as high as 50 per 1000 births were common. Now, rates of less than 5 per 1000 are often seen-more than a ten-fold reduction. Many of the interventions that prevent stillbirth, including antenatal care, hospitalization for delivery and caesarean section for foetal distress were introduced in high income countries after 1935-1940. In high income countries, it is now uncommon for stillbirths to occur at term, or intrapartum. Stillbirth rates in some low and middle income countries, and especially those with low health system coverage and quality, approximate those seen in high income countries a century ago (e.g. 30 to 50 per 1000 births) [3]. In the year 2015, there were 2.6 million stillbirths globally, with more than 7178 deaths a day. The majority of these cases occurred in developing countries. Ninety-eight percent occurred in low and middle income countries. About half of all stillbirths occurred in the intrapartum period, representing the greatest time risk. The estimated proportion of stillbirths that are intrapartum varies from 10% in developed regions to 59% in South Asia. 75% of all stillbirths in South Asia and sub-Saharan Africa, and 60% occurred in rural families from these areas. This reflects a similar distribution of maternal deaths and correlates with the areas of low-skilled health professional attendants at birth. The rate of stillbirth in sub-Saharan Africa is approximately 10 times that of developed countries, where the trends of the number of stillbirth has declined by 19.4% between 2000 and 2015, representing an annual rate of 2% [1]. There was an estimated stillbirth rate of 25.5 per 1000 births for developing countries in the year 2000, with sub-Saharan Africa representing the highest rate (32.2 per 1000 births or a total of 889,697), followed by South Asia (31.9 per 1000 or a total of 1,286,231 births, while stillbirths range from 5 per 1000 in rich countries to 32 per 1000 in South Asia and sub-Saharan Africa [4].

## Methods

**Study design:** this study was a hospital based retrospective study. Records at the maternity unit were studied dated from 1^st^ May 2014 to 30^th^ April 2017.

**Settings and population:** all women who gave birth (both through caesarean section and normal delivery) at the Buea regional hospital.

### Selection criteria

**Inclusion criteria:** all deliveries at the Buea regional hospital within the time frame. Deliveries dated from May 1^st^, 2014 to April 30^th^, 2017.

**Exclusion criteria:** transferred cases of deliveries. Incomplete documented files where not assessed.

**Sample size and sampling:** all delivery files at the Buea regional hospital dated May 1^st^, 2014 to April 30^th^, 2017. This study was a hospital based retrospective study of records at the maternity unit.

**Data collection:** the data for this study was collected using a checklist which had two parts; SHEET ONE for the outcome of all deliveries (both caesarean sections and normal delivery) and SHEET TWO for only women who had stillbirth(s). The checklist was divided into rows and columns which had demographic variables, preterm deliveries, term deliveries and post term deliveries, sex of the stillbirth, history of stillbirth, history of abortion and number of ANC's attended. As well as various age groups (<20yrs, 20-29yrs, 30-39yrs, ≥40yrs). The data obtained was tallied under the various columns with different colours of ink to indicate the various parameters under each age group. This helped facilitated the interpretation of results, for example: a preterm delivery was indicated in black ink, term deliveries in blue ink, post term deliveries in green ink and stillbirths in red ink.

$$\frac{numberofstillbirths}{numberoflivebirths+stillbirths}*1000$$

**Ethical consideration:** ethical clearance was obtained from the Faculty of Health Sciences (FHS), University of Buea and was presented at the Regional Delegation of Public Health to seek for authorization to collect the research data and it was approved. A copy of the authorization letter was presented to the director of the Buea regional hospital for approval and a letter of permission was obtained from the director as well as from the general supervisor of the Buea regional hospital.

**Data analysis:** after data was collected, it was entered into a software version where it was checked for its completeness, cleaned and analyzed accordingly. Frequency tables and graphs were used to describe some variables.

## Results

**Presentation and analysis of data:** during the period of study, there were 3577 deliveries at the Buea regional hospital, of which 93 were stillbirths giving a prevalence of 26 per thousands. With respect to age, patients were divided into four groups:< 20years; 20-29years; 30-39years; ≥40years.

**Age range of parturients:** among the 3563 parturients, of those less than 20 years (<20years): had 46(1.2%) preterm babies, 160(4.47%) term babies, and 10(0.28%) post term babies. Of those parturients between 20 and 29 years (20-29years); had 237(6.63%) preterm babies, 1812(50.66%) term babies and 66(1.85%) post-term babies. Parturients between 30-39years: had 80(2.24%) preterm babies, 1071(29.94%) term babies and 46(1.29%) post term babies; while for those ≥40years, had 6(0.16%) preterm babies, 40(1.12%) term babies and 3(0.07%) post term babies (Table 1).

**Table 1:** Gestational age versus age of parturient

		Age range				
		<20 yrs (%)	20-29 yrs (%)	30-39 yrs (%)	≥40 yrs (%)	Total
Gestational age	Preterm	46 (1.2)	237 (6.63)	80 (2.24)	6 (0.16)	369 (10.32)
	Term	160 (4.47)	1812(50.66)	1071(29.94)	40 (1.12)	3083(86.19)
	Post term	10 (0.28)	66 (1.85)	46 (1.29)	3 (0.07)	125 (3.49)

**Civil status of parturients:** two thousand and ninety one (61.49%) of the women were married and 1372 (38.51%) were unmarried giving a total of 3563 (100%) women.

**Employment status of parturients:** one thousand and fifty eight mothers were employed which gave 52.15% as compared to 1705 mothers who were unemployed which gave 47.85% (Table 2).

**Table 2:** Employment status of parturients

	Frequency	Percentage (%)
Employed	1858	52.15
unemployed	1705	47.85
Total	3563	100

**Figure 1:**
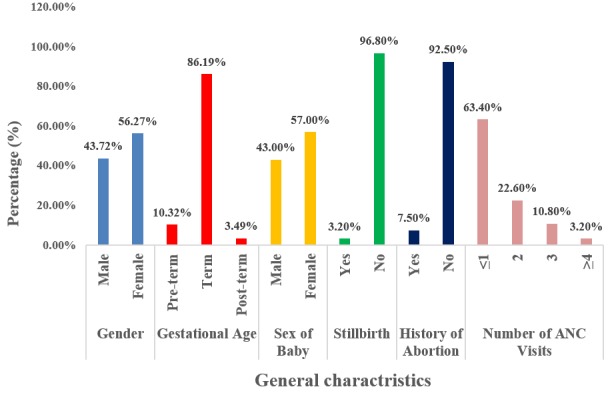
General characteristics

**Gender of the babies:** there were 2013(56.27%) females and 1564(43.72%) males of the 3577 deliveries (Figure 1).

**Gestational age for all deliveries:** among the 3577 deliveries; 369 (10.32%) were preterm, 3083 (86.19%) term and 125 (3.49%) post-term deliveries (Figure 1).

**Parturients with stillbirths:** there were 93 stillbirths, with mothers aged between 20-29years recording 57(61.3%), followed by mothers aged 30-39years, 20(21.5%) and trailed by mothers aged ≥40years 3(3.2%) (Table 3).

**Table 3:** Parturients with stillbirths

Age group of the mothers with stillbirths	Frequency	Percent (%)	Cumulative percent
<20 yrs	13	14.0	14.0%
20-29 yrs	57	61.3	75.3
30-39 yrs	20	21.5	96.8
≥40 yrs	3	3.2	100.0
Total	**93**	**100.0**	

**Time of occurrence (gestational age) of stillbirth:** stillbirth occurred most in pre-terms 56(60.2%), followed by term births - 31(33.3%) and lastly 6(6.5%) stillbirth in post-term (Table 4). 53(57%) of the stillbirths were females while 40(43%) were males (Figure 1).

**Table 4:** Time of occurrence (gestational age) of stillbirth

	Frequency	Cumulative frequency	Percent	Cumulative percent
Pre-term	56	56	60.2	60.2
Term	31	87	33.3	93.5
Post-term	6	93	6.5	100.0

**History of stillbirths:** of the 93 stillbirths, 3(3.20%) mothers had a history of stillbirth as compared to 90(96.86%) who had no history of stillbirth (Figure 1).

**History of abortion:** among the mothers who had stillbirth, 7(7.5%) have had an abortion in the past as compared to 86(92.5%) mothers who did not have an abortion (Figure 1).

**Number of antenatal clinics attended by each parturient:** fifty nine stillbirths (63.4%) was recorded by parturients with ≤1 ANC; 21 stillbirths (22.6%) recorded by parturients with 2 ANC; 10 stillbirths (10.8%) recorded by parturients with 3 ANC and 3 stillbirths (3.2%) recorded by parturients ≥4 ANC (Figure 1).

## Discussion

This study was meant to estimate the prevalence of stillbirths at the Buea regional hospital in Meme Division. The result from this study will help health care providers and the population on the prevalence of stillbirth and some possible risk factors associated with the occurrence of stillbirths in our setting. The information from this study came to the conclusion that there were 3563 parturient, those aged; less than 20 years (<20years) had 46(1.2%) preterm babies, 160(4.47%) term babies, and 10(0.28%) post term babies. Those aged between 20 and 29 years (20-29years); had 237(6.63%) preterm babies, 1812 (50.66%) term babies and 66(1.85%) post-term babies. Parturient aged between 30-39years: had 80(2.24%) preterm babies, 1071(29.94%) term babies and 46(1.29%) post term babies; while for those aged ≥40years, had 6(0.16%) preterm babies, 40(1.12%) term babies and 3(0.07%) post term babies. There were 3577 deliveries at the Buea regional hospital, of which 93 were stillbirths giving a prevalence of 26 per thousands. The stillbirth rate of 26 per thousand in this study equals that reported in the year 2009 in Cameroon [5]. Approximately equals to 25.5 per 1000 births for developing countries in the year 2000 and lower than 32.2 per 1000 births in sub-Saharan Africa in which Cameroon is inclusive [5]. It is equal to the average stillbirth rate in developing countries which has been reported to be 26 per 1000 births [6]. The stillbirth rate was significantly higher as compared to stillbirths of other developed countries such as Sweden which had a stillbirth rate of 3.6 per 1000 births [7]. The stillbirth rate of 26 per 1000 in this study is approximately five times higher than that of 5 per thousand in developed countries [6]. Stillbirths occurred most in pre-term births 56(60.2%), followed by term births 31(33.3%) and lastly 6(6.5%) stillbirths in post-term deliveries. This study is in line with results found in a journal which stipulated that stillbirth rates were high and was predominantly associated with preterm births [8]. Mothers aged between 20-29years recorded the highest rate -57(61.3%), followed by mothers aged 30-39years, 20(21.5%) and trailed by mothers aged ≥40years 3(3.2%). This is in line with results which noted that, after the introduction of routine prenatal diagnosis in McGill populations, women aged 35 years and above had fewer stillbirths as compared to their younger counterparts [9]. Furthermore, 3(3.20%) mothers had a history of stillbirth while 90(96.86%) had no history of stillbirths. This view is also shared with reports of past induced abortion, and a history of stillbirth are among other factors associated with stillbirths [6]. Also, 7(7.5%) mothers have had an abortion in the past while 86(92.5%) mothers have never had an abortion. This view is also shared with reports stated that other factors associated with stillbirths were severe anaemia, low serum folate concentration, past induced abortion, and a history of stillbirth [6].

Stillbirths occurred more in mothers who had attended fewer antenatal clinic. It occurred more in mothers who attended ≤1 ANC which was 59 stillbirths (63.4%); 2 ANC attendance which was 21 stillbirths (22.6%); 3 ANC after which accounted for 10 stillbirths (10.8%) and those with ≥4 ANC attendance had the least number of 3 stillbirths (3.2%). Furthermore, other reported risk factors for stillbirths were: maternal syphilis, maternal malnutrition; and lack of antenatal care: which improving the use of antenatal care, and nutritional status of the mother could effectively contribute towards reducing the unacceptably high burden of stillbirths in developing countries [10]. Results from this study of 53(57%) stillbirths where were females while 40(43%) were males was probably as a result of the fact that more females were delivered. Same observation was found in a research which was to determine whether the risk of stillbirths was associated with male foetal sex was modified by foetal growth >500g between 28-43weeks of gestation in Scotland between 1980 and 1996. The results concluded that male foetuses were at an increased risk of stillbirths and also there was a significant negative interaction between male and increasing birth weight in term, but not preterm deliveries [11]. Results of this study showed that stillbirths occurred more in mothers who had attended fewer antenatal clinics. It occurred more in mothers who attended ≤1 ANC which was 59 stillbirths (63.4%), those with 2 ANC attendance which was 21 stillbirths (22.6%); those with 3 ANC attendance recorded 10 stillbirths (10.8%); and those who attended ≥4 ANC had the least number of stillbirths - 3(3.2%). In developing countries risk factors of stillbirths were: maternal syphilis, maternal malnutrition; and lack of antenatal care which improving use of antenatal care, nutritional status of the mother could effectively contribute towards reducing the unacceptably high burden of stillbirths. Investigated risk factors associated with stillbirths using personal interviews and medical records abstraction in a hospital-based case control study in Thai Nguyen Province-Vietnam concluded that improved maternal health education and care in all communities may reduce the burden of foetal loss in that province which can be gotten at antenatal clinics [10,12].

**Limitation of the study:** records were not well organized, making the task of data collection difficult.

## Conclusion

It was concluded that, the prevalence of stillbirths was 26 per thousand, possible risk factors associated with stillbirth includes: the gestational age of the baby - prevalent in preterm deliveries; age (20-29years); females are more prone to stillbirths than males; a history of abortion; and insufficient ANC attendance (≤1 ANC attendance) are at high changes of having a stillbirth.

### What is known about this topic

- The prevalence of stillbirth;
- Possible risk factors associated with the occurrence of stillbirths;
- Which areas of the globe have the least or highest occurrence of stillbirths.

### What this study adds

- Educates health personnels and researchers about the prevalence of stillbirths;
- It also educates health personnels and the population at large on some possible risk factors of stillbirth and what can be done to lower it;
- It reveals an estimate on what the present occurrence of stillbirth might be in our setting and helps to fight against some cultural beliefs in our setting because when pregnant women come for ANC, they are educated for example on their feeding habits because some of them say when they eat roasted plum, it makes the baby to be dark in complexion when delivered.

## Competing interests

The authors declare no competing interests.
